# Promising role for Gc-MAF in cancer immunotherapy: from bench to bedside 

**DOI:** 10.22088/cjim.8.4.228

**Published:** 2017

**Authors:** Ehsan Saburi, Amin Saburi, Mostafa Ghanei

**Affiliations:** 1 Department of Cancer Research, Nastaran Center for Cancer Prevention, Mashhad, Iran.; 2 Department of Molecular Medicine, Zanjan University of Medical Sciences, Zanjan, Iran.; 3 Chemical Injuries Research Center, Baqiyatallah University of Medical Sciences, Tehran, Iran.

**Keywords:** Cancer, Immunotherapy, Macrophage activating factor, Gc-MAF, Vitamin D.

## Abstract

Immunotherapy has been used for years in many types of cancer therapy. Recently, cancer immunotherapy has focused on mechanisms which can enhance the development of cell-mediated immunity. Anticancer medications are administered to inhibit immunosuppressive factors such as nagalase enzyme, which is produced by neoplastic cells and destroys macrophage activating factor (Gc-MAF). Anti-neoplastics medications can also enhance immune-cell activity against tumors. Such medications show great potential in cancer immunotherapy using natural human mechanisms against neoplasms.

Cancer is one of the most frequent and important causes of mortality and morbidity in humans. In developed countries, cancer imposes a huge cost in the health system. Although cancer therapy has advanced considerably in recent decades, the cancer mortality rate has continued to rise (1). Available cancer treatments vary and include chemotherapy (mitomysin, cisplatin), radiotherapy and biological-based medications (gene therapy, RNAi treatment, hormone treatment and immunotherapy) (2-4). The current focus of drug development is on biologic drugs. These medications were designed to target the pathophysiology of cancerous cells and tissues. Angiogenesis, tumor suppression genes and surface antigens are some of the targets for these biologically-based drugs (5). Peptide immunotherapy and cell immunotherapy are two of the most interesting areas in cancer immunotherapy. T-cells, dendritic cells, natural killer cells and macrophages as immune cells are the focus of cancer immunotherapy (6). New antibodies such as bevacizumab (Avastin), which inhibits vascular endothelial growth factor (VEGF), as well as erlotinib (Tarceva), which is a kinase inhibitor that blocks epidermal growth factor receptor (EGFR) (7), have been added to chemotherapy treatments and have led to modest improvement in survival rates (6-8). These treatments relate to derivatives of cancer tissues and prevent tumor extension and spread, but the origin of the neoplastic tissues remain (9, 10). Anticancer medicines are not fully effective for the treatment of all patients. The histopathology of cancerous tissue reveals deficiencies in the defensive mechanisms of immune cells such as macrophages and neutrophils. It appears that the accumulation of these cells could be effective in the treatment of these diseases (9). Gc-MAF is a promising, new, unapproved medication as a macrophage activating factor (MAF) to treat cancer. There is solid evidence of its efficacy in cancer patients, but a number of researchers remain in doubt. The current study investigated articles containing credible scientific bases to help arrive at a proper conclusion about this product (table 1).

**Table 1:** studies on the efficacy of Gc-MAF therapy on specific cancers

	**Author**	**Cancer type**	**Gc-MAF** **dose**	**Additional therapy**	**Duration**	**Sample size**	**Follow up**	**Response**
1	Yamamoto 2008, (21)	prostate	100 (ng) IM,weekly	Nine patients received prostatectomy with or without hormone therapy. A total of 12 patients received hormone therapy	14 to 25 weeks	16	Serum Nagalase activity	no recurrence during 7 years
2	Bradstreet 2012, (41)	ASD	4 to 100 (ng) IM,weekly	none	14 weeks (±4 SD)	40	Serum Nagalase activity and Manifestations	iCGI response and decrease Nagalase activity during 100 day (Mean)
3	Thyer 2013, (43)	Cancer, MS, CFS, ALS, Autism	In various quantities and time intervals	Stem cell infusion or administration of supplements.	1 to 6 months	7	Specialized diagnostic tests	Approved treatment or improvment by specialist
4	Thyer 2013, (37)	diverse types of advanced cancers	100 (ng) IM,weekly	Complementary therapies in different patient as hormone-injections, irradiation and chemotherapy, Herceptin-therapy and others.	10 to 88 Weeks	20	Serum Nagalase activity	Approved treatment by specialist
5	Inui 2013, (36)	metastatic liver, prostate and thymus carcinoma	0.5 ml IM once or twice per week	Hyper T/NK cell therapy, Radiation, Alpha lipoic acid, High-dose vitamin C therapy, vitamin D3 and hyperthermia therapy	14 to 25 weeks	3	Specialized diagnostic tests	no recurrence during 1 years
6	Inui 2014, (35)	invasive ductal carcinoma	0.5 ml, two times a week IM	Sonodynamic therapy (SDT) and hormone therapy	3 months	1	Chest PET/CT	no recurrence almost 1 years
7	Ward 2014, (74)	advanced late stage 4 type of cancer	OA-GcMAF (880 ng) daily	Specialized Diet, D3 supplementation, low-dose acetylsalicylic acid	1 to 4 weeks	7	Serum Nagalase or anecdotic activity, Specialized diagnostic tests	decrease of tumor volume approximately 25% in a week
8	Chaiyasit 2015, (38)	Thyroid cancer	2200ng/0.5 ml IM, weekly	none	5 weeks	one	Thyroid function test ( FT3, FT4, TSH)	Reduction of CA19-9 marker, 2 years.
9	Inui 2015, (40)	pancreatic cancer (with metastasis)	colostrum MAF, 2 times, orally	none	< One month	one	Clinical signs (end stage)	weeks in coma, patient started to talk and eat
10	Inui 2015, (40)	CFS	colostrum-MAF, daily, orally	none	Few days	2	Clinical signs	significantly decreased Clinical signs
11	Inui 2016, (42)	25 yrs. history Multiple Sclerosis	0.5ml (1500 ng/0.5 ml) GcMAF, IM & SC, twiceweekly.	none	6 weeks	one	Clinical signs	walk with assistance (after 4 yrs.), Re-acquiring urinary control
12	Inui 2016, (39)	Non-small Cell Lung Cancer (stage 3B)	0.5 ml (1500 ng/0.5 ml), two times a week, IM	SDT, TTF & ozone therapy	9 months	one	neuron-specific enolase (NSE) & contrast-enhanced computed tomography (CT)	decreased to normal range (<16.3ng/ml)

**Function, biological and pharmacological properties of Gc-globulin and Gc-MAF: **The vitamin D-binding protein (DBP) Gc-globulin (human group-specific component), in addition to the storage and transport of vitamin D, has an important physiological function as a scavenger of extracellular G-actin to increase neutrophil chemotaxis and macrophage activation (11). Studies have shown that Gc- globulin is a protein with a structure similar to albumin that is a receptor of active vitamin D3 (12). Gc- globulin plays a role in immune system regulation, osteoclastic activity and as a primary defense against infectious factors such as immunodeficiency virus and sepsis. Gc-globulin when modified is capable of affecting the activation and fortification of immune cells exhibiting anticancer activity. These molecules activate macrophages after deglycosylation through β-galactosidase and sialidase of the B and T lymphocytes, respectively. This product supports phagocyte, superoxidase and immunopotentiatory activity of Gc-MAF (group specific component-macrophage activating factor; fig 1) (11-13). 

Macrophages activated by Gc-MAF offer different properties that are effective against a variety of cancers in human and animal models (14, 15). Moreover, phenotypes of Gc-globulin influence the MAF levels in serum (16) and activate mouse peritoneal macrophages (17). Previous studies have confirmed that Gc as a precursor to Gc-MAF is considerably non-specific or even completely deglycosylated in cancer patients (fig 2) (18-20). 

**Figure 1:**
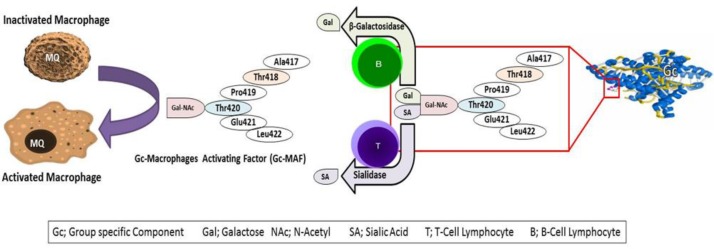
Gc-MAF Generation Cascade (11, 14)

**Figure 2:**
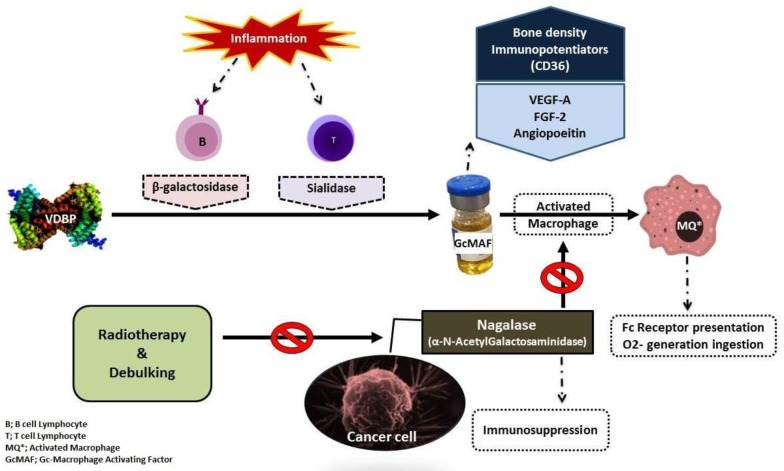
The mechanism of Gc-MAF activities against neoplasm (8, 16).

MAF precursor activity has also been lost or reduced after Gc-globulin treatment in some cancer cell lines. This appears to result from the deglycosylated ɑ-N-acetylgalactosaminidase (nagalase) secreted from cancerous cells (18). Nagalase has been detected in many cancer patients, but not in healthy individuals (19). Studies have shown that the production of nagalase has a mutual relationship with Gc-MAF level and immunosuppression (14, 19, 21). It has been demonstrated that serum levels of nagalase are good prognosticators of some types of cancer (22). The nagalase level in serum correlates with tumor burden and it has been shown that Gc-MAF therapy progresses, nagalase activity decreases (21). 

**Gc-MAF and Angiogenesis: **One aspect of cancer immunotherapy is angiogenesis inhibition. Angiogenesis is an agent in cancer progression and many chemotherapy agents or immunotherapies have been suggested for its inhibition (23-25). It has been shown that Gc-MAF can inhibit the angiogenesis induced by pro-inflammatory prostaglandin E1 (26). It stimulates 3'-5'-cyclic adenosine monophosphate (cAMP) formation in human peripheral blood mononuclear cells (PBMCs) (27) and mediates it through the CD36 receptor (28). The effect of Gc-MAF on chemotaxis or activation of tumoricidal macrophages is likely the main mechanism against angiogenesis. 

Gc-MAF for treatment of cancerous cells in vitro: Inflammation induced by an adjuvant is critical to decreasing the time required for and optimization and development of immune response (29). Phagocytosis and antigens are present in inflammation-primed macrophages and are crucial to the development of antibodies facilitated by Gc-MAF as an adjuvant. Administration of Gc-MAF stimulates immune-cell progenitors for extensive mitogenesis, activates macrophages and produces antibodies. “This indicates that Gc-MAF is a powerful adjuvant for immunization.” Cancer cell lines do not develop into tumor genes in mouse models after Gc-MAF-primed immunization (29-31) and the effect of Gc-MAF has been approved for macrophage stimulation for angiogenesis, proliferation, migration and metastatic inhibition on tumors induced by MCF-7 human breast cancer cell line (15, 32). 

A previous study has verified Gc-MAF treatment in autism-derived macrophages moderates’ dysregulated gene expression (33) and is the same effect to counteract the neurotoxicity of oxaliplatin, a cancer chemotherapy drug (34).

Gc-MAF f**or treatment of cancer: **Inui et al. reported that Gc-MAF resulted in the complete disappearance of all symptoms and complications in a 55-year-old female with breast cancer presenting as a right axillary tumor, spinal metastasis, intrapleural nodular tumors and right pleural effusion. The protocol included: "a high dose of second-generation Gc-MAF (0.5 ml) administered twice a week intramuscularly for a total of 21 injections.” Complementary therapies such as sonodynamic therapy (SDT) were used to obtain the best results. In less than a year, a significant increase in monocyte percentage along with a rapid decrease in tumor markers with no serious side effects were observed (35). 

Yamamoto et al. showed that the administration of Gc-MAF to 16 patients with prostate cancer led to improvements in all patients without recurrence (21). Inui et al. reported that a 74-year-old man diagnosed with prostate cancer with multiple bone metastases was in complete remission nine months after initiation of GcMAF therapy simultaneously with hyper T/NK cell, high-dose vitamin C and alpha lipoic acid therapy (36). Thyer et al. reported that three men with prostate cancer showed increased nagalase serum levels. About one year after the onset of treatment with GcMAF, they showed a significant decrease in serum nagalase activity (37). One study evaluated a decrease in Gc-MAF precursor activity in oral cancer patients with squamous cell carcinoma (SCC) and patients with pancreatic cancer and showed an enhancement of the immune system (22). Inui et al. studied cancer immunotherapy with second-generation Gc-MAF (36). In addition to Gc-MAF, they administered immune cells, alpha lipoic acid, vitamin C and vitamin D3 alternatively (36). Gc-MAF has been verified for use in colon, thyroid (38), lung (39), liver, thymus (36), pancreatic (40), bladder and ovarian cancer and tongue squamous carcinoma (37). It has also been approved for non-neoplastic diseases such as autism (41), multiple sclerosis (42, 43), chronic fatigue syndrome (CFS) (40), juvenile osteoporosis (44) and systemic lupus erythematous (45).

**Patient Selection: **It important to know which patients and which types of cancers are the best candidates for Gc-MAF therapy. Prostate, breast, colon, liver, stomach, lung (including mesothelioma), kidney, bladder, uterus, ovarian, head/neck and brain cancers, fibrosarcomas and melanomas are the types of cancer tested thus far (37). The tumoricidal response to Gc-MAF therapy of cancer depends on its type and current stage. Primarily as cell membrane antigens were presented to activated macrophages, weekly administration of 100 ng Gc-MAF to cancer at different stages and types showed curative effects at different follow-up times (30, 46). 

Gc-MAF therapy has been reported to be more efficient and rapid for treatment of undifferentiated tumor cells such as adenocarcinoma of the breast and prostate cancer cells, than with differentiated cells such as squamous carcinoma cells. This curative rate appears to depend on both the number of receptors on a particular antigen for macrophages and the quantity of antigens present in each cell (37). Anemia and other hematologic disorders can confound the efficacy of Gc-MAF; therefore, this treatment has been suggested for non-anemic patients. In vitro studies have shown that human prostate and breast cancer cells are sensitive to the effects of Gc-MAF independently of macrophage activation (15, 47). In these studies, cancer cells treated in vitro with Gc-MAF seemed to have the effects of their neoplastic phenotype and metastatic potential, thus, providing a biological basis to explain the results obtained in patients harboring prostate and breast cancer (11, 12).

**Dosage, frequency and treatment duration of Gc-MAF administration: **It is important to determine the dosage of Gc-MAF, the frequency of administration and the length of time required for response to therapy before decisions about its administration can be made. Studies have shown that weekly administration of 100 ng Gc-MAF to cancer patients had curative effects on a variety of cancers (30, 46). Because the half-life of the activated macrophages is approximately one week, it must be administered weekly. In animal studies, the dosage for administration of Gc-MAF for macrophage activation is considered to be 20-100 pg/mouse (48). 

In vitro activated human macrophages with Gc-MAF (100 pg/ml) killed 60% and 86% of MCF-7 breast cancer cell lines at an effector/target ratio of 1.5 after 4 h and 18 h of incubation, respectively. Hence, the potency of Gc-MAF is 1,000-fold higher than that of lyso-alkylglycerol (BCG-tumor)-derived MAF. In vivo weekly intramuscular administration of Gc-MAF (100 ng) for 16-22 weeks was used to treat patients with breast cancer (21, 35-37, 46, 49). 

It is likely that the frequency of administration will depend upon the individual responsiveness associated with vitamin D receptor (VDR) polymorphism. The association between polymorphisms of gene coding for VDR should be taken into account. Differential responses to Gc-MAF have been observed in human monocytes (26) as well as in metastatic breast cancer (50) These studies demonstrated that individuals harboring different VDR genotypes had different responses to Gc-MAF and that some genotypes were more responsive than others. It could be proposed that the dose and length of treatment should be altered according to the individual VDR genotype. This observation raises the issue of molecular interaction between Gc-MAF and VDR.

There is evidence that the ability of VDR to translocate to the outer plasma membrane induces an unspecific and rapid function in the interaction with vitamin D (51, 52). Molecular alignment between the amino terminus of the Gc-MAF and carboxyl terminus of the VDR (37) is suggested as the interaction site. Oleic acid an unsaturated fatty acid, bound to Gc-MAF and stabilize and facilitate translocation of the complex in the plasma membrane (53, 54). It seems Gc-MAF and VDR have multiple sites of interaction that suggest direct interaction without the need for vitamin D, Gc-globulin along with vitamin D can be internalized in cells and the interaction with VDR may occur more strongly and longer intracellularly (11, 55, 56).

It has been demonstrated that macrophages activated by Gc-MAF bind immediately (less than 30 min) to neoplastic prostate, breast, colon, oral and ovarian cells in vitro, but there is a lack of clinical evidence about the action of activated macrophages after Gc-MAF administration to cancerous cells (21) Also, 48 h post-administration of 50 pg Gc-MAF to mice, a large number of antibody secreting cells were observed by Jerne plaque assays (48). 

It has been reported that heat-killed Ehrlich ascites tumor cells were no longer transplantable into mice after six days of treatment with Gc-MAF (29). Furthermore, it should also be considered that the response of the organism harboring a cancer has to take an important factor into account when it comes to immunotherapy with Gc-MAF. In fact, the role of systemic inflammation and VDR gene polymorphisms in the prognosis of advanced cancer patients was recently demonstrated (57). 

**Adjunct medications: **In some studies, adjunct drugs (INF-α and anti-thrombin-3) or supplemental immunotherapy (NK/T cell therapy) has been used to increase the efficiency of Gc-MAF (36, 58). Gc-MAF can be effective in the prevention of lesion progression through accompanying factors like VEGF-A, FGF-2 and CD36 receptor-mediated angiopoietin (27, 28). The role of chemotherapy medications in conjunction with Gc-MAF therapy must be determined. Administration of Gc-MAF for cancer patients exclusively activates macrophages as an important cell in adaptive immunity. It has been demonstrated that Gc-MAF does not directly activate other immune cells, such as dendritic cells (35), but that contributing macrophages to processed antigens via MHC-II antigen complex-mediation to T-cells appear to predominantly engaged B cells (21). Gc-MAF supports humoral immunity by producing, developing and releasing large quantities of antibodies against cancer. Clinical evidence from a human model of breast cancer patients supports this hypothesis (36, 37). There is also evidence that confirms the tumoricidal role of Gc-MAF via Fc-receptor mediation (59).

When exploring the role of other types of chemotherapy in conjunction with Gc-MAF, the role of nutrition cannot be overlooked. A favorable PINI score is associated with prolonged survival of advanced cancer patients and it is logical to assume that cancer patients who can prevent the onset or presence of cachexia will have a much stronger response to immunotherapy with Gc-MAF (60). The complexity of changes to the immune system in response to chronic inflammation associated with cachexia is far greater than previously envisaged. It is likely that the best therapeutic responses will be observed when the nutritional and inflammatory aspects are taken together with stimulation of the immune system (61).

**Side effects: **Side effects of chemotherapy in cancer is an important complication. Although the optimized anti-cancer activity of Gc-MAF occurs at 100 ng/human with a 30-fold increase in the digestion index of phagocytic capacity and a 15-fold increase in the superoxide generating capacity of peripheral blood adhering monocytes (macrophages), it appears that the administration of more than 10 times of this dose range for a 3-6 month period is safe (48, 62). The natural activation mechanism of macrophages by Gc-MAF is so natural and it should not have any side effects on humans or animal models even in cell culture (15, 37, 47, 63). In addition to these considerations, it should be noted that no harmful side effects of Gc-MAF treatment have been reported, even when it was successfully administered to autistic children (64). 

**Markers for response to treatment: **Information from previous clinical investigations on the efficacy of Gc-MAF evaluated the response to treatment using clinical methods (such as measurement of nagalase level). Other valid methods are required to prove the claim of “being cancer-free during the follow-up period” such as a histopathologic study. Evidence demonstrates that persistent serum levels of PSA at 24 weeks after Gc-MAF therapy might suggest the opposite (21). Besides the Gc-MAF efficacy on macrophage activity, it can be a potential anti-angiogenic agent (28) and an inhibitor of the migration of cancerous cells in the absence of macrophages (47). 

**Future use for cancer treatment: **Although many types of cancer are the focus for Gc-MAF therapy, it has not been used as a clinical treatment for lung and brain cancer. In initial study showed an antitumor effect of degalactosylated gc-globulin on orthotopic grafted lung cancer in mice (65). Gc-MAF was assessed for salivary gland adenocarcinoma cell-derived nagalase and researchers concluded that the “data strongly suggests that HSG alpha-nagalase acts as an immunodeficiency factor in cancer patients” (20). Facial neoplasms can be a future target for Gc-MAF therapy.

Articles on cancer immunotherapy focus on medications that can inhibit the spread of neoplasms by activating immune cells such as T-cell lymphocytes and natural killer cells. Toll-like receptor agonists, dendritic cell-based treatments and microorganism-based vaccines such as Bacillus Calmette-Guerin (BCG) have been suggested for cancer immunotherapy (5, 66). Activating or modifying natural killer cells, dendritic cells, DC, CTL, INF and IL-2 have all been recommended for cancer immunotherapy (67). Most available immune-based medications for oncotherapy perform a specific function such as inhibiting angiogenesis or inducing apoptosis, but the goal is to find a medication that can cover all these therapeutic aspects. Immunotherapy for cancer should preferably have greater efficacy with fewer adverse effects. This review focused on a new medication which is often neglected in cancer immunotherapy projects and surveys. Discussions currently disregard immune cell activation against cancer Gc-globulin and Gc-MAF. Clinical investigations have confirmed Gc-MAF efficacy on cancerous patients. Although Gc-MAF is not a routine medication in cancer immunotherapy, the mechanism of this medication has been fully elucidated.

Inflamed tissues, such as those infected or aggressed tissue by neoplastic cells, release cell membrane lipid metabolites such as lysophosphotydylcholine (lyso-Pc) (67-69). Gc-globulin as a DBP is required for activation of macrophages. When neoplastic tissue is present, Gc-globulin as a precursor can be hydrolyzed and activated with the sialidase of T lymphocytes and β-galactosidase of lyso-Pc-primed B lymphocytes in stepwise fashion to yield the potent MAF (11). This results in the conversion of Gc-globulin to Gc-MAF, which activates macrophages to phagocyte neoplastic cells. When macrophages cannot be properly activated, the infection and neoplasms can form an aggressive tumor (11).

 If an external source can provide the required amount of Gc-MAF to activate macrophages, phagocytosis is initiated and the tumor will decrease. It has been reported that nagalase cannot deglycosylate Gc-MAF as it has specificity for Gc globulin alone (70). 

Several reports have agreed upon the mechanism of Gc-MAF function. It was demonstrated that inflammation-derived macrophage activation with the participation of B and T lymphocytes is the main mechanism (71). Intra-tumoral administration of BCG or other bacterial cells through induced inflammation in cancerous tissues can lead to regression of local and metastasized tumors (72). These reports clarified the development of specific immunity against tumors and provided the term tumor-associated antigen (TAA); however, induced inflammation by BCG in non-cancerous normal tissue has been shown to have no effect on the neoplastic tissue (72). Inflamed (BCG treated) tumor tissue is able to release tumor cell membrane lipid metabolites such as alkylglycerols and lyso-alkylphospholipids because neoplastic cell membranes contain alkylphospholipids (35). Both alkylglycerols and lyso-alkylphospholipids can act as macrophage-activating agents and are at least 400 times more potent than lysophospholipids. This indicates that macrophages highly-activated by the addition of Gc-MAF can show tumoricidal activity. This may explain the reason for inflammation having a direct effect on limiting cancerous tissue (35). 

Previous clinical investigations have confirmed the efficacy of Gc-MAF. In addition to activating existing macrophages, Gc-MAF is a potent mitogenic factor that can stimulate the myeloid progenitor cells to increase systemic macrophage cell counts by 40-fold in four days (48). The recent availability of food-based Gc-MAF, which is Gc-MAF produced during the fermentation of milk products, can provide further fields of research and application of this promising approach (73). 

Although Gc-MAF was successfully used for immunotherapy of cancer patients, their aspect should be considered for future research.

In conclusion activation and contribution of cells (NK and T helper lymphocytes) and factors relating to immunotherapy are more complicated and costly than using Gc-MAF therapy. Moreover, these cells and factors have shown about 10-fold lower potent activity than the naturally activated (inflammation-primed) macrophages. There is a need to design further studies to directly compare the efficacy of routine cancer immunotherapy using activating NK versus Gc-MAF therapy.

 The question must also be posed as to why this medication has not yet been approved by the FDA. Despite the doubts raised as results of some clinical studies, the efficacy of this drug has been endorsed in several studies. It appears that there are non-scientific reasons that prevent FDA approval.

## Abbreviation

**ASD**; Autism spectrum disorders,** BCG**; Bacillus Calmette-Guerin, **cAMP**; 3'-5'-cyclic adenosine mono phosphate, **CFS**: chronic fatigue syndrome, **EGFR**; Epidermal Growth Factor Receptor, **FT3**; Free tri-iodothyronine, **FT4**; free thyroxine, **Gc**; Group specific component, **Gc-MAF**; Group specific component-Macrophage Activating Factor, **HCT116**; colorectal cancer cell line**, HepG2**; human hepatoma cell line, **IM**; intramuscularly, **INF-α**; interferon-α, **lyso-Pc**; lyso phosphotydyl choline, **MAF**; Macrophage Activating Factor, **MCF-7**; human breast cancer cell line, **MS**; Multiple Sclerosis, **Nagalase**; α-N-Acetylgalactosaminidase, **NK cell**; natural killer cell, **PBMCs**; peripheral blood mononuclear cells, **RNAi**; RNA interfering, **SC**; subcutaneously, **SCC**; squamous cell carcinoma, **SDT**; Sonodynamic Therapy, **SRBC**; sheep red blood cells, **TSH**; thyroid stimulating hormone, **VDBP=DBP**; Vitamin D Binding Protein, **VDR**; vitamin D receptor, **VEGF**; Vascular Endothelial Growth Factor.
